# Supplementary material for: Risk Factors for COVID-19 in Inflammatory Bowel Disease: A National, ENEIDA-Based Case–Control Study (COVID-19-EII)
Source: J Clin Med. 2022 Dec 19;11(24):7540. doi: 10.3390/jcm11247540 (PMC9785640; doi:10.3390/jcm11247540)
Supplement: Supplementary file 1 [file jcm-11-07540-s001.zip › jcm-1949196-supplementary.pdf]

Supplementary Table S1. Comorbidities between cases and controls

**Variable**	**Cases** ***n* = 482**	**Controls** ***n* = 964**	**Univariate** ***P*- value**
Arterial hypertension, *n* (%)	107 (22)	160 (17)	0.39
Dyslipidaemia, *n* (%)	74 (15)	115 (12)	0.70
Immune-mediated diseases, *n* (%)	51 (10.5)	83 (8.6)	0.39
Diabetes mellitus, *n* (%)	36 (7.5)	63 (6.5)	0.64
Ischemic cardiomyopathy, *n* (%)	31 (6.4)	50 (5.2)	0.99
Cardiac insufficiency, *n* (%)	8 (1.7)	6 (0.6)	0.12
Chronic renal insufficiency, *n* (%)	19 (3.9)	20 (2)	0.17
Chronic obstructive pulmonary disease, *n* (%)	17 (3.5)	23 (2.3)	0.61
Neoplasia, *n* (%)	17 (3.5)	33 (3.4)	0.92
Cirrhosis, *n* (%)	6 (1.2)	5 (0.5)	0.43
Cerebral stroke, *n* (%)	5 (1)	15 (1.5)	0.19
Dementia, *n* (%)	4 (0.8)	1 (0.1)	**0.08**
Comorbidities, *n* (%)			
None	276 (57)	620 (64)	**0.01**
One	111 (23)	186 (19)	0.12
Two or more	95 (20)	158 (16)	0.12
Charlson score, *n* (%)			**0.02**
Zero	173 (36)	376 (39)	
Mild (one-two)	195 (40)	404 (42)	
Moderate (three-four)	66 (14)	130 (13)	
Severe (five or more)	48 (9.9)	53 (5.5)	
